# Combined Omeprazole and *Glycyrrhiza glabra* L. Extract Attenuate Ethanol-Induced Gastric Ulceration Through Modulation of TLR4/NF-κB/NLRP3 Signaling and Upregulation of PI3K/AKT/mTOR Gene Expression

**DOI:** 10.3390/ijms27157037

**Published:** 2026-08-05

**Authors:** Sahar Khateeb, Mody Albalawi, Amnah Obidan, Fahad M. Almutairi, Hanan Abdulrahman Sagini, Eman F. S. Taha

**Affiliations:** 1 Department of Biochemistry, Faculty of Science, University of Tabuk, Tabuk 71491, Saudi Arabia; 2 Biochemistry Division, Department of Chemistry, Faculty of Science, Fayoum University, Fayoum 63514, Egypt; 3 Department of Biological Sciences, College of Sciences, University of Jeddah, Jeddah 21959, Saudi Arabia; 4 Health Radiation Research Department, National Center for Radiation Research and Technology, Egyptian Atomic Energy Authority (EAEA), Cairo 11787, Egypt

**Keywords:** *Glycyrrhiza glabra* L., ulcer, omeprazole, oxidative stress, inflammation

## Abstract

Ethanol (EtOH)-induced gastric ulcer (GU) is a common model used to investigate mechanisms of mucosal injury and repair. Omeprazole (OMP) is a conventional anti-ulcer drug that effectively suppresses gastric acid secretion, but its efficacy may be enhanced by combining it with bioactive phytochemicals derived from a *Glycyrrhiza glabra* L. (licorice; LIQ) extract that possess potent antioxidant and anti-inflammatory properties. The aim of the present study was to evaluate the gastroprotective effects of OMP, LIQ extract, and their combined treatment against EtOH-induced GU in rats, focusing on modulation of TLR4/NF-κB/NLRP3 signaling and PI3K/AKT/mTOR gene expression. The ethanolic extract of LIQ was chemically characterized by LC-ESI-QTOF-MS/MS, and molecular docking was performed to evaluate the potential binding interactions of its major constituents with H^+^/K^+^-ATPase and COX-2. Thirty male Wistar rats were randomly allocated into control, ulcer (ULC), OMP-treated, LIQ-treated, and combined treatment groups. GU was induced by absolute EtOH. Subsequently, gastric pH, stomach coefficient, oxidative stress, inflammatory mediators, and PI3K, AKT, and mTOR gene expression were assessed. Histopathological and immunohistochemical analyses of mucosal architecture and the expression of TNF-α, caspase-3, and PCNA were performed. Coadministration of OMP and LIQ demonstrated the greatest gastroprotective activity, marked by a significant increase in gastric pH, restoration of antioxidant status, and substantial reduction in ROS, TLR4, NF-κB, and NLRP3 levels. The combined therapy significantly upregulated the expression of PI3K, AKT, and mTOR genes in comparison to ULC. Histopathological and immunohistochemical findings further demonstrated preservation of gastric mucosal integrity, reduced inflammatory cell infiltration, and decreased TNF-α, caspase-3, and PCNA immunoreactivity, indicating attenuation of mucosal injury. In conclusion, LIQ extract enhanced the gastroprotective effect of OMP against EtOH-induced GU, supporting its potential as an adjunct to OMP. Further studies are warranted to confirm the underlying molecular mechanisms.

## 1. Introduction

A gastric ulcer (GU) is a prevalent gastrointestinal disorder characterized by a lesion in the stomach lining that may extend into the submucosa or muscularis propria [1]. GU is associated with significant complications, including perforation, hemorrhage, and obstruction, which contribute to increased morbidity and mortality [2]. It has been identified as a consequence of an imbalance between destructive and protective factors of gastric mucosal layers through various endogenous pathways [3]. This imbalance is due to many variables, including excessive alcohol intake, pathogenic infections, stress, and non-steroidal anti-inflammatory medicines [4]. Ethanol (EtOH) is a prevalent addictive substance, and its overconsumption is associated with the development of GU. Therefore, EtOH has been widely used as an ulcerogenic agent in GU models. EtOH consumption induces structural and functional disruptions in the gastric mucosal barrier, which is characterized by decreased endogenous antioxidant defenses with an increased generation of reactive oxygen species (ROS). This diminishes antioxidant capability, leading to oxidative stress and apoptosis [5,6].

Proton pump inhibitors (PPIs) are potent therapeutic agents widely employed as the primary treatment for GU [7,8]. Omeprazole (OMP) is one of the most commonly prescribed PPIs in clinical practice; however, the use of OMP is limited by low efficacy against GU and several potential adverse effects [9,10]. Hence, there is a growing interest in finding safer and more effective gastroprotective medicines. Traditional medicines, encompassing medicinal plants and herbal therapies, are widely employed as alternative remedies for various health issues, including gastrointestinal disorders [11,12]. Biogenic sources, including terpenoids, alkaloids, and phenolics, encompass bioactive compounds recognized for their significant antioxidant, anti-inflammatory, and antiapoptotic properties, potentially providing considerable protection to the gastrointestinal system [13,14]. Consequently, these natural resources can be utilized to develop innovative therapies for the enhancement of health [15].

*Glycyrrhiza glabra* L. (licorice; LIQ) extract has been extensively examined for its diverse pharmacological properties, which include anti-ulcer, anti-inflammatory, antioxidant, and antiviral effects, in addition to its protective function in the gastrointestinal system [6,16]. The Toll-like receptor 4 (TLR4)/nuclear factor kappa B (NF-κB)/NOD-like receptor family pyrin domain-containing 3 (NLRP3) signaling pathway is closely involved in the inflammatory response associated with EtOH-induced GU [17]. Furthermore, the phosphoinositide 3-kinase (PI3K)/protein kinase B (AKT)/mechanistic target of rapamycin (mTOR) signaling pathway plays a vital role in preserving gastric epithelial cell integrity and enhancing mucosal healing, and its activation has been linked to protection against EtOH-induced GU [18]. Nonetheless, the gastroprotective potential of combined LIQ and OMP therapy in EtOH-induced GU has not yet been investigated. Moreover, it remains unclear whether any potential protective effects of this combination are associated with coordinated modulation of the inflammatory and pro-survival signaling pathways. Accordingly, the present study investigated the gastroprotective efficacy of OMP, LIQ, and their combined administration against EtOH-induced GU in rats, with particular emphasis on the underlying anti-inflammatory, antioxidant, anti-apoptotic, and molecular mechanisms involving TLR4/NF-κB/NLRP3 signaling and PI3K/AKT/mTOR gene expression.

## 2. Results and Discussion

### 2.1. DPPH Radical Scavenging Activity

**Figure 1:**
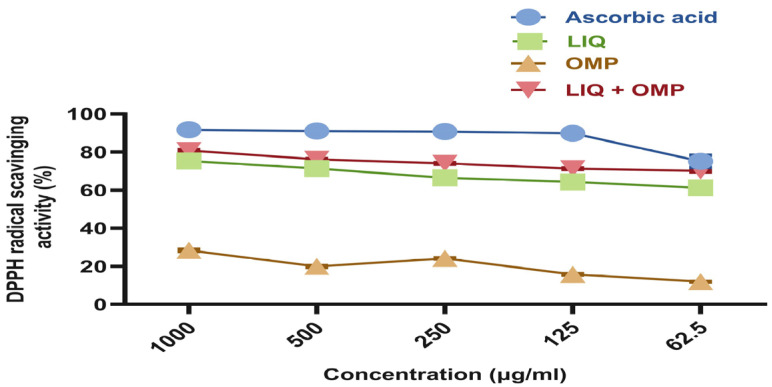
DPPH radical scavenging activity (%) of LIQ extract, OMP, LIQ + OMP, and ascorbic acid. Values are presented as mean ± SD (*n* = 3).

As shown in Figure 1, ascorbic acid exhibited strong antioxidant activity, with inhibition percentages exceeding 90% at concentrations ≥ 250 µg/mL and remaining above 70% at 62.5 µg/mL. The LIQ extract demonstrated a dose-dependent antioxidant effect, with % inhibition ranging from 75.63 ± 0.28 at 1000 µg/mL to 61.08 ± 0.48 at 62.5 µg/mL. However, OMP alone exhibited relatively low antioxidant activity, with % inhibition not exceeding 30% at the highest concentration tested (28.45 ± 0.60 at 1000 µg/mL) and dropping to 11.92 ± 0.15 at 62.5 µg/mL, indicating limited radical scavenging capacity. The combination of LIQ and OMP displayed improved antioxidant activity compared to OMP alone. At 1000 µg/mL, the combination showed 80.72 ± 0.55% inhibition, which is slightly higher than LIQ alone. The findings reveal that the LIQ extract is the main contributor to antioxidant activity and suggest that the phenolic compounds in the extract are crucial for free radical scavenging, as noted in prior studies [19]. This enhanced antioxidant capacity may, therefore, potentiate the gastroprotective efficacy of OMP.

### 2.2. LC–ESI–QTOF–MS/MS Characterization of LIQ Extract

**Figure 2:**
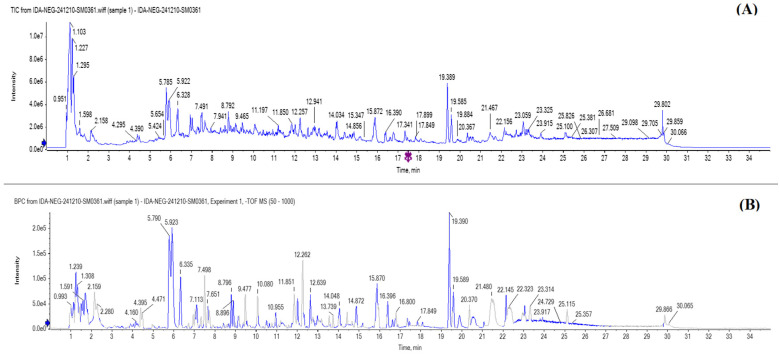
LC–ESI–QTOF–MS chromatographic profiles of LIQ extract acquired in negative ionization mode. (**A**) Total ion chromatogram (TIC) and (**B**) base peak chromatogram (BPC).

The acquired total ion chromatogram (TIC) and base peak chromatogram (BPC) exhibited a chemically varied metabolomic profile, encompassing flavonoids, chalcones, and triterpenoid saponins as displayed in Figure 2.

Eighteen metabolites were tentatively identified (Table 1) using accurate mass measurements, MS/MS fragmentation patterns, and comparison with published literature. These metabolites represented the major phytochemical constituents of *Glycyrrhiza glabra*. The characteristic triterpenoid saponin glycyrrhizic acid was detected at RT 14.56187 min with a deprotonated molecular ion [M–H]^−^ at *m*/*z* 821.4034, while its aglycone glycyrrhetinic acid was observed at RT 19.3861 min with [M–H]^−^ at *m*/*z* 470.3405. Several glycosylated flavonoids were identified, including quercitrin, baicalein-O-glucuronide, Kaempferol-O-pentoside, and quercetin-O-pentoside, along with daidzein-8-C-glucoside, an isoflavone C-glycoside. A quinic acid derivative, chlorogenic acid, was also identified among the detected metabolites. In addition, the isoflavones daidzein and formononetin, the flavones luteolin and apigenin, and the flavanones liquiritigenin and naringenin were identified, supporting the established antioxidant and pharmacological potential of the extract. The detection of licochalcone A and glabridin further substantiated the presence of prenylated flavonoid derivatives known for their anti-inflammatory and cytoprotective properties. The presence of these bioactive constituents may contribute to the gastroprotective potential of LIQ extract [20].

**Figure 3:**
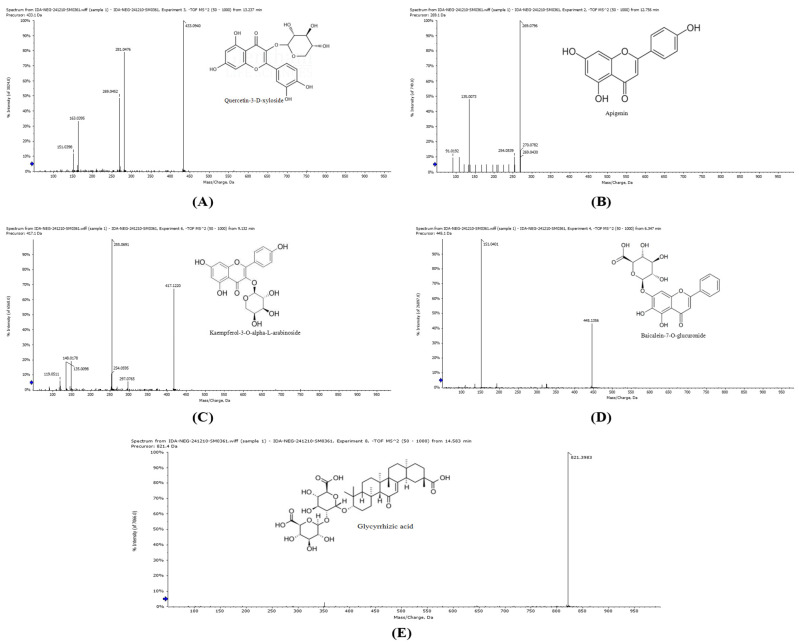
Chemical structures of selected representative metabolites tentatively identified in LIQ extract: (**A**) Quercetin-O-pentoside, (**B**) Apigenin, (**C**) Kaempferol-O-pentoside, (**D**) Baicalein-O-glucuronide, and (**E**) Glycyrrhizic acid.

Five representative metabolites were selected for detailed structural visualization based on chromatographic abundance, spectral quality, and phytochemical relevance, representing diverse metabolite classes identified in the extract (Figure 3). Furthermore, selected metabolites with documented biological activities were subjected to molecular docking analysis to evaluate their potential interactions with the investigated biological targets.

### 2.3. Molecular Docking Results

Molecular docking of glycyrrhizic acid and quercetin-O-pentoside was carried out at the binding site of H^+^/K^+^-ATPase, yielding docking scores of −8.04 and −7.33 kcal/mol, respectively. Both compounds may interact favorably with H^+^/K^+^-ATPase by binding to the active site of the target protein. Compared with the standard anti-ulcer drug OMP, which showed a docking score of −5.31 kcal/mol, both compounds demonstrated more favorable docking scores (Table 2).

**Figure 4:**
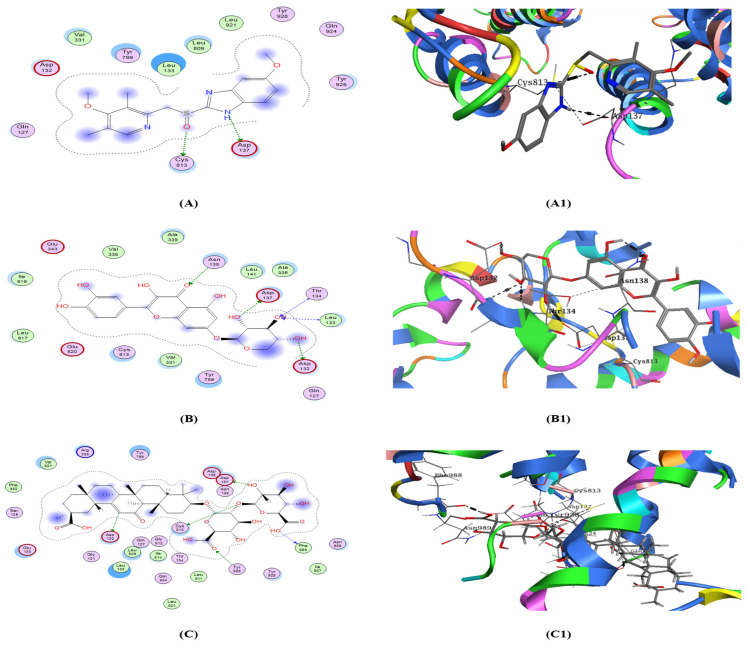
2D (left panels) and 3D (right panels) binding interactions of (**A**,**A1**) OMP, (**B**,**B1**) Quercetin-O-pentoside, and (**C**,**C1**) glycyrrhizic acid within the active site of H^+^/K^+^-ATPase.

As shown in Figure 4, glycyrrhizic acid and quercetin-O-pentoside displayed five H- bonds with Cys 813, Asp 137, Tyr 928, Phe 988, Asp 132, Asn 138, Thr 134 and Leu 133 compared to OMP that displayed two H-bonds with Cys 813, Asp 137. Furthermore, the bond length of H-bond for the docked ligands falls within the range of 3.97 to 2.58 Å compared to that of OMP from 3.35 to 3.05 Å that potentiates the binding interaction of glycyrrhizic acid and quercetin-O-pentoside and may contribute to binding stability. Moreover, the extra hydrophobic interactions of glycyrrhizic acid and quercetin-O-pentoside render their binding within the core of the active site similar to that of the reference ligand.

**Figure 5:**
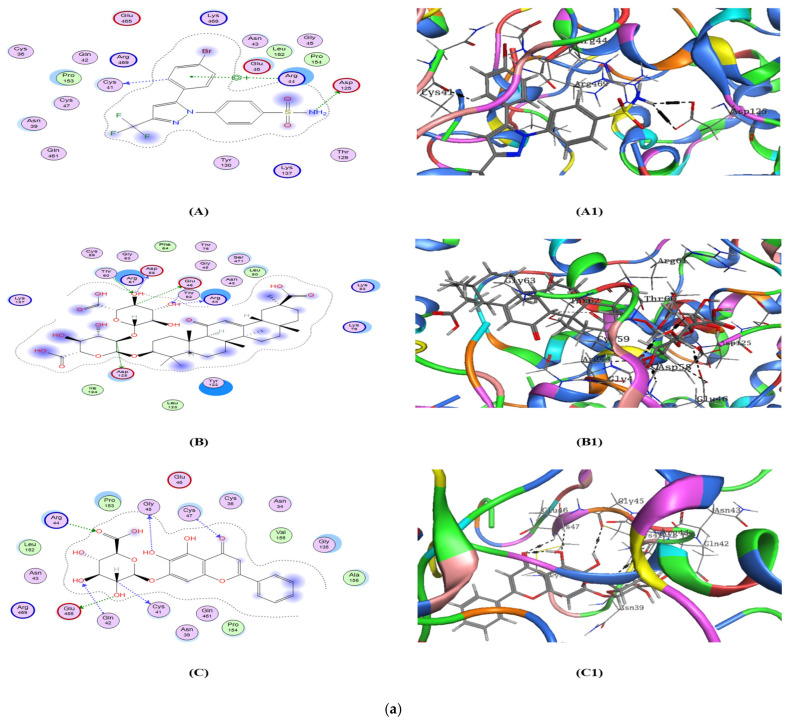

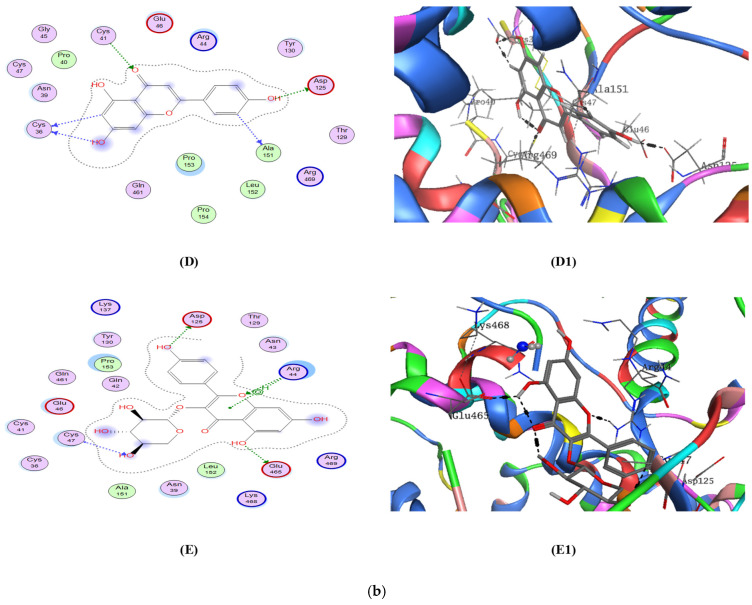
(**a**) 2D (left panels) and 3D (right panels) binding interactions of (**A**,**A1**) Bromo celecoxib, (**B**,**B1**) Glycyrrhizic acid, and (**C**,**C1**) Baicalein-O-glucuronide within the active site of COX-2. (**b**) 2D (left panels) and 3D (right panels) binding interactions of (**D**,**D1**) Apigenin and (**E**,**E1**) Kaempferol-O-pentoside within the active site of COX-2.

Docking results of glycyrrhizic acid, Baicalein-O-glucuronide, apigenin, Kaempferol-O-pentoside, and standard drug bromo celecoxib (SC-558) against cyclooxygenase-2 (COX-2) revealed that all tested compounds were well-fitted and properly oriented within the binding pocket of the COX-2 (Table 3). Interestingly, glycyrrhizic acid and baicalein-O-glucuronide showed favorable predicted binding in the COX-2 binding site with superior docking score of −8.20 and −7.89 kcal/mol, respectively. Furthermore, as displayed in Figure 5a, glycyrrhizic acid showed six H-bond interactions, two of them with Asp 125 (2.76 Å) and Arg (2.47 Å) compared to a bromo celecoxib interaction that binds to Asp 125 (3.23 Å) and Arg (4.96 Å). Similarly, baicalein-O-glucuronide exhibited six H-bond interactions, two of them with Arg 44 (2.21 Å) and Cys 41 (2.80 Å) compared to bromo celecoxib interaction that exhibited an arene-H bond interaction with Arg (4.96 Å) and H-bond interaction with Cys 41 (2.86 Å). On the other hand, studying the binding mode of apigenin and Kaempferol-O-pentoside indicated that they were able to form five and four H-bond interactions, respectively (Figure 5b). For the docked compounds, the bond length is shorter than that formed with bromo celecoxib and there was an extra hydrophobic bond interaction; thus, the tested compounds exhibited favorable predicted binding affinity in COX-2 binding.

### 2.4. Final Body Weight, Stomach Weight, Stomach Coefficient, and Gastric pH Evaluation

**Figure 6:**
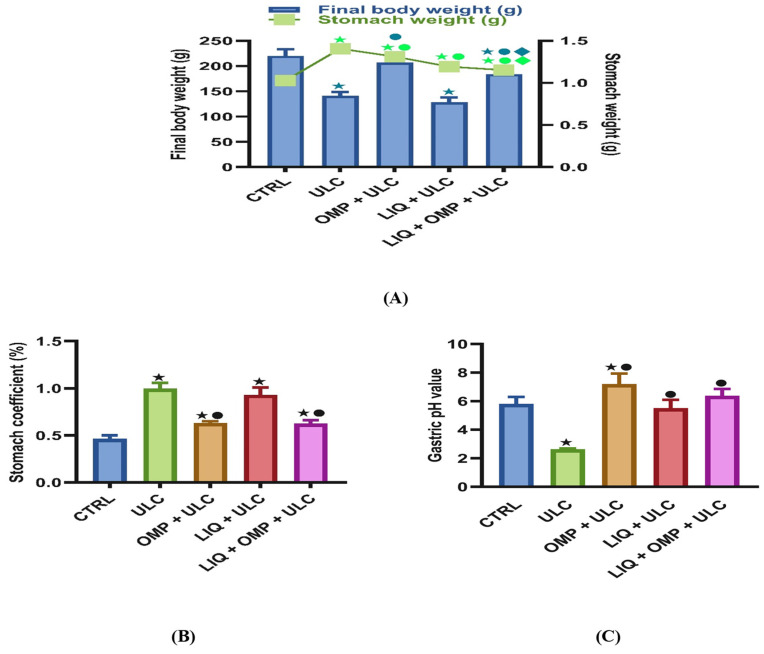
Effect of OMP, LIQ, and their Combination on (**A**) final body weight and stomach weight, (**B**) stomach coefficient, and (**C**) gastric pH in EtOH-induced GU in rats. Data are presented as mean ± SD. Statistical analysis was performed using One-way ANOVA followed by Tukey’s post hoc test. Statistical significance was defined as *p* < 0.05. Symbols indicate comparisons as follows: (★) versus CTRL, (●) versus ULC, and (◆) versus OMP + ULC.

As illustrated in Figure 6, the final body weight in the ulcer (ULC) group was significantly reduced (by 35.9%) compared to the control (CTRL) group. The decrease in body weight indicates a detrimental effect of EtOH exposure, which may be related to gastric mucosal injury and inflammation. Similarly, Albalawi and Khateeb [21] observed declines in body weight following stomach damage caused by EtOH. Conversely, the body weight was significantly increased after treatment with OMP, with an approximately 47% increase relative to the ULC group, but was statistically nonsignificant compared to CTRL (*p* = 0.1170). The treatment with LIQ showed a lesser improvement, still significantly lower than CTRL (−41.6%) and not significantly different from ULC (*p* = 0.1493). The combination treatment markedly improved body weight, with an approximately 30.3% increase relative to the ULC group and a decrease of 16.5% compared to CTRL (*p* < 0.0001). Overall, OMP, either alone or in combination with LIQ, alleviated the detrimental impact of EtOH on body weight.

The ULC group showed a significant increase in stomach weight (36.7%) and stomach coefficient (114.3%) compared with CTRL, which may be attributed to gastric edema/inflammation and a significant decline in body weight. These alterations may indicate variations in gastric motility and inflammation, which are crucial in EtOH ulceration [22,23,24]. On the other hand, treatment with OMP, LIQ, and their combination significantly reduced stomach weight compared with the ULC group, showing reductions of 6.6%, 15.1%, and 18.0%, respectively. Likewise, OMP and the combination treatment significantly reduced the stomach coefficient by 36.5% and 37.2%, respectively, relative to the ULC group, while LIQ alone showed only partial, nonsignificant improvement (*p* = 0.1761). The reductions in stomach weight and coefficient suggest attenuation of gastric inflammation and restoration of mucosal integrity.

Moreover, the ULC group exhibited a significant reduction in gastric pH (2.633 ± 0.0516) compared to the CTRL (5.817 ± 0.4750), consistent with previous findings [25]. The gastric pH was significantly elevated in the OMP group (pH: 7.200 ± 0.7294) compared to both the ULC group and the CTRL group, indicating its potent acid-suppressive effect as a PPI [26]. In contrast, treatment with LIQ moderately increased gastric pH (5.517 ± 0.5776) relative to the ULC group, though not significantly different from the CTRL (*p* = 0.8463), suggesting a protective rather than acid-suppressive role. Interestingly, the combination therapy group demonstrated an elevation in pH to 6.383 ± 0.4665, significantly higher than the ULC group, though not significantly different from treatment with LIQ or OMP individually. These effects may be attributed to the recognized anti-ulcer, anti-inflammatory, and antioxidant properties of LIQ and its bioactive constituents [6,16]. Overall, these findings support the potent acid-reducing capacity of OMP and the gastroprotective effect of LIQ, with their combination offering complementary benefits in mitigating EtOH-induced gastric acidity.

### 2.5. Effect of Treatments on Ulcer Index (UI) and Ulcer Inhibition

The UI was used to assess the severity of gastric mucosal damage (Table 4). The ULC group exhibited a markedly elevated UI (19.65 ± 0.09), confirming successful induction of GU when compared with the CTRL group, which showed no ulcerative lesions. Treatment with OMP resulted in a moderate reduction in ulcer severity, as evidenced by a decreased UI (16.64 ± 0.14), corresponding to an ulcer inhibition of 16.46 ± 0.04%. A more pronounced gastroprotective effect was observed in animals treated with LIQ, which showed a significantly lower UI (10.53 ± 0.03) and a higher ulcer inhibition percentage (46.69 ± 0.17). Notably, combined treatment with LIQ and OMP produced the greatest protective effect against gastric mucosal injury, reducing the UI to 1.65 ± 0.19 and achieving the highest ulcer inhibition rate (90.61 ± 0.05%). Overall, these findings demonstrate that LIQ exhibits significant anti-ulcer activity, which is markedly enhanced when combined with OMP, resulting in substantial attenuation of ulcer severity.

### 2.6. Macroscopic and Histopathological Evaluation of Gastric Mucosa

**Figure 7:**
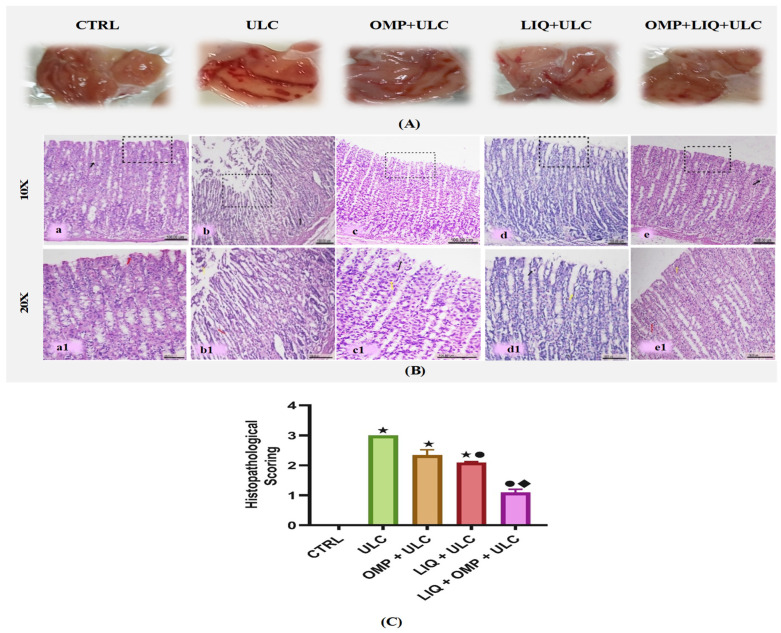
Macroscopic and histopathological evaluation of gastric mucosa in the experimental groups. (**A**) Macroscopic photographs of the gastric mucosa lesions. (**B**) Histopathological examination of gastric mucosa and submucosa layers showing: (**a**) normal fundic mucosa (black square), gastric pits (black arrow) and (**a1**) normal epithelial layer (red arrow); (**b**) sloughing of the mucosa (black square) degenerated gastric gland (black arrow), (**b1**) cell debris (yellow arrow) and inflammatory cells (red arrow); (**c**) superficial regeneration of lamina epithelialize (black square), (**c1**) with vacuolar degeneration of simple columnar cells (black arrow) and intact lamina propria (yellow arrow); (**d**) normal architectures of tunica mucosa (black square), (**d1**) vacuolation of epithelial cells (black arrow) and cell necrosis (yellow arrow); (**e**) intact gastric mucosa (black square) and area of necrosis (black arrow), (**e1**) superficial regeneration (yellow arrow) and inflammatory cells (red arrow). (H&E, Scale bar: 100 µm). (**C**) Histopathological injury scores. Statistical significance was defined as *p* < 0.05. Symbols indicate comparisons as follows: (★) versus CTRL, (●) versus ULC, and (◆) versus OMP + ULC.

Macroscopic examination of the stomach in experimental groups revealed variable alterations. The ULC group exhibited extensive ulcerative lesions, edema, and hemorrhagic areas, reflecting severe damage to the stomach mucosa, consistent with previous reports [27,28,29]. On the other hand, treatment with OMP, LIQ, and their combination diminished these macroscopic lesions, which indicates a gastroprotective effect (Figure 7A). Moreover, the histopathological assessment of the gastric mucosa and submucosa layers in the CTRL group revealed normal fundic mucosa, gastric pits, and an intact epithelial layer (Figure 7B(a,a1)). In contrast, the ULC group exhibited erosion of the gastric mucosa and a loss of architectural structure in the surface mucous epithelium, along with sloughing of the mucosa and significant infiltration of inflammatory cells (Figure 7B(b)). Additionally, there was a noticeable sloughing of the gastric gland cells, with the presence of degenerated cells that lacked nuclei (Figure 7B(b1)).

The OMP + ULC group showed moderate improvement of tunica mucosa characterized by vacuolar degeneration of simple columnar cells and superficial regeneration of lamina epithelialize (Figure 7B(c,c1)). In the LIQ + ULC group, the gastric fundus showed normal architecture of the tunica mucosa with sloughing of mucous neck cells and moderate inflammatory cell infiltration (Figure 7B(d,d1)). Moreover, the LIQ + OMP + ULC group exhibited a significant improvement in histological abnormalities, such as the restoration of normal structures of the mucosa layer and normal gastric glands with low infiltration of inflammatory cells (Figure 7B(e,e1)). Furthermore, histopathological damage scores exhibited significant differences across the experimental groups (Kruskal–Wallis, *p* < 0.05). The ULC group exhibited the highest tissue damage score, whereas all treatment groups showed reduced histopathological severity. The combined treatment LIQ + OMP produced the lowest histopathological score among the ulcer-induced groups, indicating the greatest protective effect, and differed significantly from both ULC (*p* < 0.0001) and OMP + ULC group (*p* < 0.05) (Figure 7C).

### 2.7. Effects of Treatments on Renal and Liver Function Biomarkers

EtOH-induced GU markedly elevated liver and kidney biomarker levels in the ULC group relative to CTRL, indicating hepatic and renal dysfunction [30]. Treatment with OMP or LIQ significantly ameliorated these alterations compared with the ULC group. The combined treatment (LIQ + OMP) exhibited the greatest improvement, showing significantly lower levels of liver and renal biomarkers than the ULC group and either monotherapy group (Table 5). These findings suggest superior hepatorenal protective effects of the combined treatment, likely due to the antioxidant and anti-inflammatory characteristics of *Glycyrrhiza glabra*, which may enhance the cytoprotective action of OMP [31,32].

### 2.8. Impact of Treatments on Oxidative Stress and Antioxidant Biomarkers

**Figure 8:**
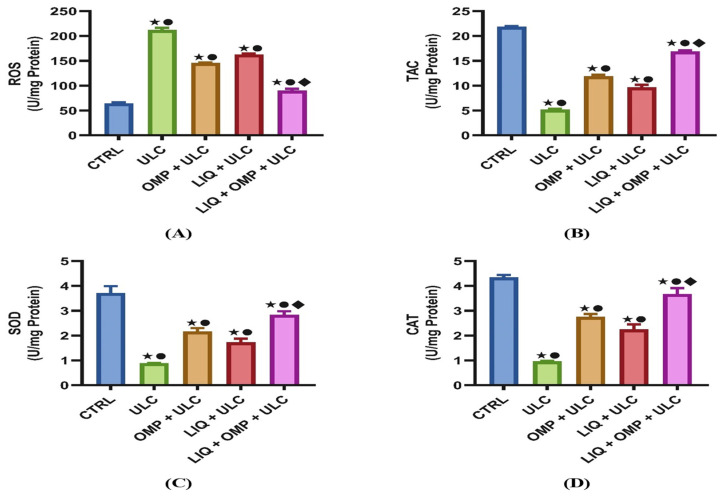
Oxidative stress and antioxidant biomarkers (**A**) ROS, (**B**) TAC, (**C**) SOD, and (**D**) CAT in different experimental groups. Data are presented as mean ± SD. Statistical analysis was performed using One-way ANOVA followed by Tukey’s post hoc test. Statistical significance was defined as *p* < 0.05. Symbols indicate comparisons as follows: (★) versus CTRL, (●) versus ULC, and (◆) versus OMP + ULC.

In comparison to the CTRL group, the ULC group demonstrated a significant increase in ROS levels, with a 228% increase (*p* < 0.0001). This finding was accompanied by a substantial reduction in total antioxidant capacity (TAC) (76.2%, *p* < 0.0001), along with decreases in superoxide dismutase (SOD) and catalase (CAT) activity by 76% and 77.7%, respectively (*p* < 0.0001), showing compromised antioxidant defenses following EtOH-induced GU (Figure 8). These results align with other research indicating that EtOH increases oxidative stress by elevating ROS generation and diminishing antioxidant enzyme activities [13,33]. Excessive formation of ROS induces lipid peroxidation and cellular damage, resulting in the depletion of endogenous antioxidants and the disruption of cellular defense mechanisms [34,35,36].

Conversely, OMP treatment markedly reduced ROS levels by 31.3%, while increasing TAC by 128.9%, restoring SOD levels by 144%, and enhancing CAT levels by 184% compared to the ULC group (*p* < 0.0001), consistent with previous findings [37]. Furthermore, LIQ treatment markedly reduced ROS levels by 22.9% (*p* < 0.0001), increased TAC by 86.4% (*p* < 0.0001), reinstated SOD levels by 95.5% (*p* < 0.0005), and elevated CAT levels by 132.9%, compared to ULC (*p* < 0.0001). The combination treatment (LIQ + OMP) resulted in a significant reduction in ROS, with a 57.5% decrease, restored TAC levels with a 224.5% increase, enhanced SOD by 219%, and achieved the maximum recovery of CAT activity, with a 279.4% increase compared to the ULC group (*p* < 0.0001) (Figure 8). Therefore, the co-administration of OMP and LIQ provided enhanced protection against gastric damage, since OMP diminishes gastric acid secretion while LIQ enhances antioxidant and anti-inflammatory responses. Furthermore, these results were supported by the in vitro DPPH assay, which demonstrated enhanced free radical scavenging efficacy of the LIQ–OMP combination. These findings corroborate previous research demonstrating the antioxidant effects of LIQ extract and its capacity to diminish oxidative stress and free radical generation [38]. The antioxidant properties of LIQ are attributed to its abundant bioactive phytochemicals, which exhibit potent antioxidant, anti-inflammatory, and anti-ulcer effects [39,40].

### 2.9. Impact of Treatments on TLR4/NF-κB/NLRP3 Signaling Pathway

**Figure 9:**
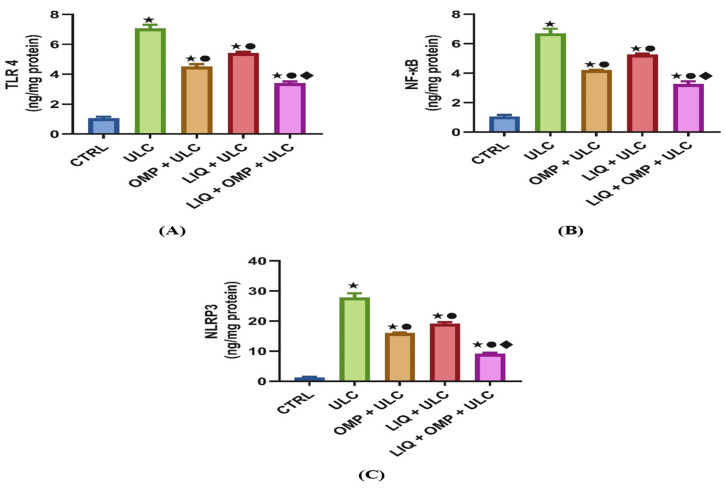
Effects of OMP, LIQ extract, and their combination on gastric levels of (**A**) TLR4, (**B**) NF-κB, and (**C**) NLRP3 in EtOH-induced GU in rats. Data are presented as mean ± SD. Statistical analysis was performed using One-way ANOVA followed by Tukey’s post hoc test. Statistical significance was defined as *p* < 0.05. Symbols indicate comparisons as follows: (★) versus CTRL, (●) versus ULC, and (◆) versus OMP + ULC.

A significant increase in TLR4/NF-κB/NLRP3 expression levels was observed in the ULC group relative to the CTRL group (*p* < 0.0001), with TLR4 expression upregulated by 563%, NF-κB by 533%, and NLRP3 by 2001% (Figure 9). These results align with earlier studies suggesting that the TLR4/NF-κB/NLRP3 signaling pathway is crucial in the inflammatory response associated with EtOH-induced GU [17]. Activation of TLR4 initiates NF-κB signaling and subsequently activates the NLRP3 inflammasome, which enhances inflammatory responses and causes gastric damage [41]. NF-κB serves as a principal regulator of inflammatory gene expression in gastric epithelial cells and is activated by many stimuli, including ROS, leading to the production of pro-inflammatory cytokines such as TNF-α [34,42]. Prior research has associated NF-κB activation with the development of GU [43]. Furthermore, NLRP3 is a pivotal intracellular inflammasome involved in the development of gastric injury, and inhibiting its activation has been demonstrated to mitigate EtOH-induced stomach damage [17,44].

The administration of OMP reduced the levels of TLR4 by 36%, NF-κB by 37%, and NLRP3 by 42% compared to the ULC group (*p* < 0.0001). These findings are consistent with those reported by Alzokaky et al. [45], who showed that OMP markedly reduced NF-κB p65 and NLRP3 levels relative to the ULC group. Similarly, LIQ extract resulted in a 23% decrease in TLR4, a 22% drop in NF-κB, and a 31% decline in NLRP3 levels relative to the ULC group (*p* < 0.0001). The combination therapy (LIQ + OMP) produced the most significant reductions, demonstrating a 52% decrease in TLR4, a 51% reduction in NF-κB, and a 67% decrease in NLRP3 levels relative to the ULC group (*p* < 0.0001). The combination treatment showed substantial differences when compared to OMP treatment alone (Figure 9). These results align with other studies indicating that LIQ extract inhibits TLR4/NF-κB activation [46]. Furthermore, bioactive constituents of LIQ have been reported to inhibit TLR4 signaling and significantly suppress the activation of the NLRP3 inflammasome [47,48], whereas glycyrrhizin demonstrates substantial anti-inflammatory effects by modulating NF-κB and NLRP3 signaling pathways [49]. These findings indicate that the combined administration of OMP and LIQ may mitigate stomach damage in GU, potentially through modulation of the TLR4/NF-κB/NLRP3 pathway. This effect may reflect the complementary mechanisms of both agents: OMP provides anti-inflammatory effects, while LIQ bioactive constituents exert antioxidant and inflammasome-modulating actions.

### 2.10. Effect of Treatments on PI3K, AKT, and mTOR Gene Expression

**Figure 10:**
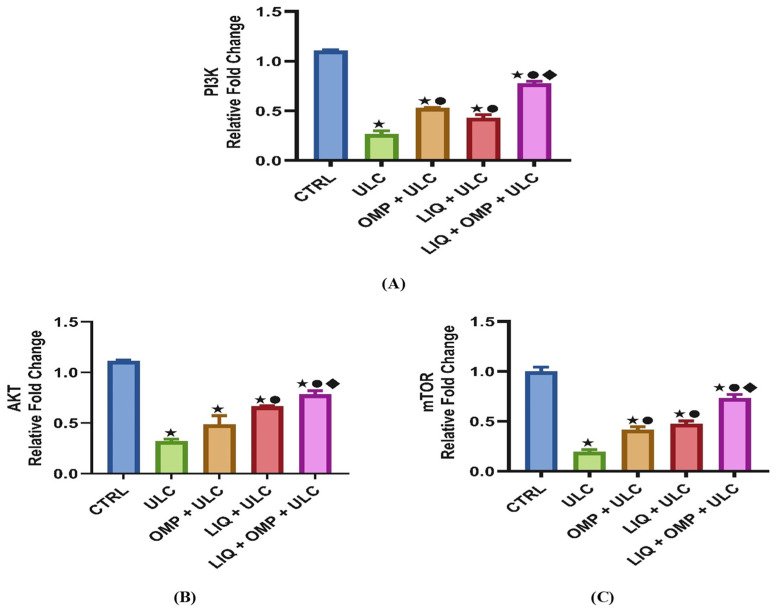
Effect of Treatments on (**A**) PI3K, (**B**) AKT, (**C**) and mTOR gene expression in EtOH-induced GU in rats. Data are presented as mean ± SD. Statistical analysis was performed using One-way ANOVA followed by Tukey’s post hoc test. Statistical significance was defined as *p* < 0.05. Symbols indicate comparisons as follows: (★) versus CTRL, (●) versus ULC, and (◆) versus OMP + ULC.

The PI3K/AKT/mTOR signaling pathway regulates key cellular processes, including cell proliferation, apoptosis, and inflammation. Dysregulation of this pathway has been implicated in the occurrence and progression of diseases [50,51]. The current investigation showed that EtOH-induced GU resulted in a significant downregulation of PI3K, AKT, and mTOR gene expression levels by 73.3%, 67.9%, and 80.4%, respectively, in the ULC group compared to the CTRL group (*p* < 0.0001) (Figure 10), consistent with previous findings [18]. Compared with the ULC group, OMP increased PI3K, AKT, and mTOR expression by 98.7%, 52.1%, and 112%, respectively. However, AKT expression in the OMP-treated group did not show a significant difference when compared to the ULC group (*p* = 0.0646). Likewise, LIQ therapy enhanced the PI3K, AKT, and mTOR gene expression levels by 61.3%, 106.8%, and 141.8%, respectively, in comparison to the ULC group.

Combined treatment with LIQ and OMP exhibited the greatest improvement in restoring PI3K, AKT, and mTOR gene expression, with increases of 189.2%, 143.5%, and 272.9%, respectively, relative to the ULC group. Furthermore, PI3K and mTOR expression levels were significantly higher in the combination group than in either the OMP or LIQ monotherapy groups, indicating greater upregulation of PI3K and mTOR gene expression following combined treatment. LIQ extract and its bioactive constituents have been reported to activate the PI3K/AKT signaling pathway and inhibit apoptosis, thus contributing to the protection of EtOH-induced GU [52,53]. Thus, the improved therapeutic effect observed with the combined treatment may be associated with modulation of PI3K, AKT, and mTOR gene expression, together with the complementary antioxidant, anti-inflammatory, and anti-apoptotic properties of LIQ and OMP. However, further protein-level studies are required to confirm pathway modulation.

### 2.11. Immunohistochemical (IHC) Evaluation

**Figure 11:**
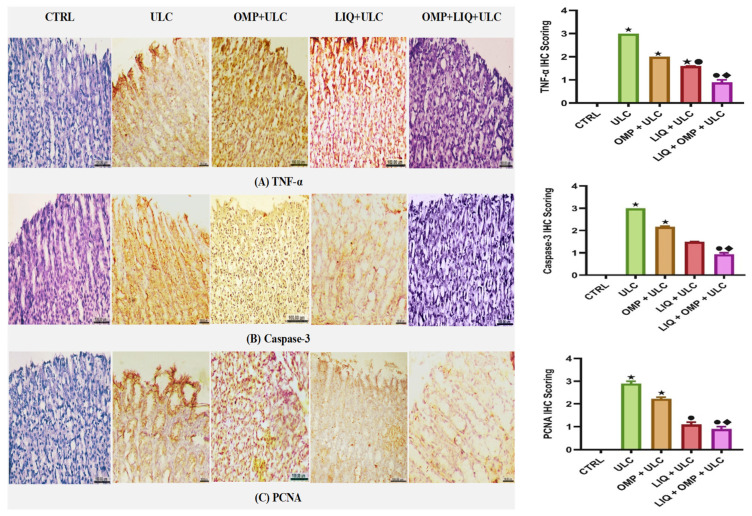
Immunohistochemical expression of TNF-α, Caspase-3, and PCNA and semi-quantitative IHC scores among the experimental groups. (Scale bar: 100 µm). Group differences were analyzed using the Kruskal–Wallis test followed by Dunn’s post hoc test. Statistical significance was defined as *p* < 0.05. Symbols indicate comparisons as follows: (★) versus CTRL, (●) versus ULC, and (◆) versus OMP + ULC.

The IHC expression of tumor necrosis factor-α (TNF-α), caspase-3, and proliferating cell nuclear antigen (PCNA) was assessed as an indicator of inflammation, apoptosis, and cellular proliferation to further elucidate the mechanisms of the gastroprotective effects of LIQ and OMP. TNF-α, caspase-3, and PCNA exhibited the highest expression levels in the ULC group, indicating pronounced inflammatory, apoptotic responses, and suggesting enhanced regenerative and DNA repair response to mucosal injury (Figure 11). These findings support the important role of inflammation in EtOH-induced GU, where disruption of the gastric mucosal barrier promotes inflammatory responses characterized by increased TNF-α production and activation of NF-κB signaling, thereby aggravating ulcer progression [5,54,55]. Caspase-3 is a crucial effector protein in the apoptotic pathway, and its elevated expression has been documented in experimental GU models, indicating increased apoptotic cell death [56,57]. PCNA serves as a recognized marker for cellular proliferation and DNA replication, which are critical for the healing of gastrointestinal ulcers [58]. Elevated PCNA expression and immunoreactivity have been previously documented in experimentally produced GUs [59,60], consistent with the present findings. The elevated PCNA expression in the ULC group likely reflects a compensatory response to mucosal injury rather than effective mucosal regeneration, consistent with the concurrent downregulation of PI3K/AKT/mTOR gene expression.

Compared with the ULC group, TNF-α, caspase-3, and PCNA were decreased in the OMP + ULC group, but the differences were not statistically significant (TNF-α, *p* = 0.7261; Caspase-3, *p* > 0.9999; PCNA, *p* > 0.9999). In the LIQ + ULC group, TNF-α and PCNA exhibited considerable reductions (TNF-α, *p* = 0.0122; PCNA, *p* = 0.0188), whereas caspase-3 showed a non-significant decline relative to the ULC group (*p* = 0.3156). The combined OMP + LIQ treatment produced the greatest improvement, partially restoring these markers toward normal levels (Figure 11), with significant reductions compared with the ULC group (TNF-α, *p* < 0.0001; caspase-3, *p* = 0.0001; PCNA, *p* = 0.0004), suggesting attenuation of inflammation and apoptosis, along with reduced compensatory proliferation. These findings are supported by prior research indicating that LIQ and its bioactive constituents exhibit anti-inflammatory and anti-apoptotic activities that are associated with reduced TNF-α expression, modulation of NF-κB-related inflammatory signaling, decreased caspase-3 activity, and attenuation of oxidative stress [61,62,63,64,65]. Similarly, OMP has been shown to mitigate gastric inflammation by diminishing TNF-α expression and lowering PCNA expression in experimental GU models, thus enhancing its gastroprotective effects [66]. The diminished PCNA expression noted in the treated groups may indicate a reduction in mucosal damage and a restoration of gastric tissue integrity.

### 2.12. Correlation Analysis

**Figure 12:**
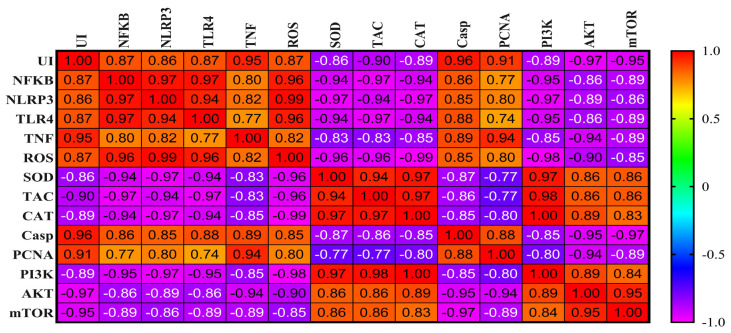
Heatmap illustrating Spearman’s rank correlation coefficients among inflammatory, oxidative stress, apoptotic, proliferative, and survival signaling biomarkers. Positive correlations are represented by lighter colors, whereas negative correlations are represented by darker colors. Correlation strength is expressed as Spearman’s correlation coefficient (ρ).

Spearman’s rank correlation analysis showed the presence of a clear clustering pattern amongst the examined biomarkers (Figure 12). Highly positive correlations were observed between UI, NF-κB, NLRP3, TLR4, TNF-α, ROS, caspase-3 and PCNA confirming a strong association between inflammation, oxidative stress, apoptotic activity and tissue injury progression. In contrast, antioxidant markers (SOD, TAC, and CAT) and PI3K/AKT/mTOR signaling molecules were significantly positively correlated with one another and negatively correlated with inflammatory and oxidative stress parameters. These data suggest that the augmentation of antioxidant defense and the upregulation of PI3K, AKT, and mTOR gene expression are strongly associated with the downregulation of inflammatory signals and apoptotic responses. Collectively, the correlation analysis supports the proposed mechanistic function of oxidative stress-mediated inflammatory signaling in GU disorder and highlights the involvement of the PI3K, AKT, and mTOR genes in mediating the observed treatment effects.

### 2.13. Study Limitations

The present study has several limitations. The phytochemical characterization of the LIQ extract was qualitative, and the metabolites were tentatively identified by LC-ESI-QTOF-MS/MS using accurate mass measurements, MS/MS fragmentation patterns, and literature comparisons, without quantitative standardization or confirmation using authentic reference standards. Only a single dose and treatment duration were evaluated for the combined LIQ and OMP therapy, and potential pharmacokinetic interactions between LIQ and OMP were not investigated. Furthermore, the PI3K/AKT/mTOR pathway was assessed only at the mRNA level without protein-level validation. Future studies should include quantitative phytochemical standardization, pharmacokinetic evaluation, dose optimization, and protein-level validation to further strengthen the mechanistic understanding and translational relevance of these findings.

## 3. Materials and Methods

### 3.1. Materials and Preparation of LIQ Extract

OMP powder was obtained from Sigma-Aldrich (St. Louis, MO, USA). Roots of *Glycyrrhiza glabra* L. were obtained from a commercial herbal market in Egypt and taxonomically verified by specialists in the Department of Botany, Faculty of Agriculture, Zagazig University, Egypt, according to the Egyptian Drug Authority Community Herbal Monograph for *Glycyrrhiza glabra* (EMA/HMPC/571119/2023). The dried roots of *Glycyrrhiza glabra* L. were ground into a fine powder. Briefly, 100 g of the powdered material was macerated in 500 mL of 80% (*v*/*v*) ethanol at 4 °C overnight [67]. The suspension was subsequently agitated on an orbital shaker at 150 rpm for 24 h. Following extraction, the mixture was filtered using Whatman No. 1 filter paper, and the filtrate was concentrated under decreased pressure using a rotary evaporator (BUCHI Labortechnik AG, Flawil, Switzerland) at 40 °C. The resulting crude extract yielded 16.8% (*w*/*w*), based on the initial dry plant material. The extract was transferred to an airtight amber container and stored at 4 °C until further analysis. A single extraction batch was prepared and used for all subsequent phytochemical and biological experiments to ensure experimental consistency.

### 3.2. DPPH Radical Scavenging Assay

The free radical scavenging activity of LIQ extract, OMP, LIQ + OMP, and ascorbic acid was evaluated using the DPPH assay according to Fu et al. [68], with minor modifications. Samples were dissolved in methanol to obtain final concentrations of 62.5, 125, 250, 500, and 1000 μg/mL. An aliquot (100 μL) of each sample was mixed with 100 μL of 0.1 mM DPPH. OMP and LIQ stock solutions were prepared at same concentrations and mixed at a 1:1 (*v*/*v*) ratio immediately before the assay. The mixtures were incubated in darkness for 30 min at ambient temperature, and the absorbance was assessed at 517 nm utilizing a microplate reader (Multiskan Go, Thermo Fisher Scientific Oy, Vantaa, Finland). The blank consisted of methanol without DPPH, whereas the negative control comprised methanol containing a DPPH solution. The DPPH radical scavenging activity percentage was determined using the formula:

DPPH scavenging (%) = {(A_control_ − A_sample_)/A_control_} × 100 (1)

where A_control_ and A_sample_ are the absorbances of the control and the sample, respectively.

### 3.3. Tentative Identification of LIQ Metabolites by LC–ESI–QTOF–MS/MS

Metabolites in the LIQ extract were tentatively identified using LC–ESI–QTOF–MS/MS. Briefly, 50 mg of the dried extract was dissolved in 1 mL of a water/methanol/acetonitrile mixture (50:25:25 *v*/*v*/*v*), vortexed for 2 min, sonicated for 10 min, then centrifuged at 10,000 rpm for 10 min. The supernatant was diluted with the same solvent mixture to obtain the working analytical solution. A procedural blank was prepared and analyzed under identical conditions. An aliquot (10 μL) was injected into the LC–MS/MS system. Chromatographic separation was performed using an ExionLC™ UPLC system (SCIEX, Concord, ON, Canada) equipped with an XSelect HSS T3 C18 analytical column (2.1 × 150 mm, 2.5 μm, 100 Å; Waters Corporation, Milford, MA, USA). The column was maintained at 40 °C, and the flow rate was 0.30 mL/min. For analysis in the negative ionization mode, the mobile phase comprised a 5 mM ammonium formate buffer (pH 8.0, adjusted with 1 N NaOH) containing 1% methanol (mobile phase B) and 100% acetonitrile (mobile phase C). The gradient elution program was as follows: 0–1 min, 5% solvent C; 1–21 min, 5–95% solvent C; 21–28 min, 95% solvent C; 28–28.1 min, 95–5% solvent C; and 28.1–35 min, 5% solvent C.

MS detection was conducted utilizing a TripleTOF^®^ 5600+ mass spectrometer (SCIEX, Concord, ON, Canada) equipped with an electrospray ionization (ESI) source operating in the negative ion mode. Full-scan TOF-MS data were obtained across an *m*/*z* range of 50–1000, subsequently followed by information-dependent acquisition (IDA) for MS/MS analysis. The ion source parameters were as follows: ion spray voltage, −4500 V; curtain gas (CUR), 25 psi; ion source gas 1 (GS1), 45 psi; ion source gas 2 (GS2), 45 psi; source temperature (TEM), 500 °C; and collision energy (CE), −35 eV. For IDA, precursor ions exceeding 200 counts per second (cps) were selected for fragmentation, with a maximum of 15 candidate ions monitored per acquisition cycle. Previously fragmented precursor ions were excluded after three repeated acquisitions for 3 s, using an isotope exclusion window of 2.0 Da and a mass tolerance of 10 ppm. Dynamic background subtraction was enabled throughout data acquisition. Raw LC–MS/MS data were processed using MS-DIAL version 4.9, PeakView™ (Version 1.2), and MasterView™ software for peak detection, alignment, spectral deconvolution, and MS/MS spectral interpretation. Metabolites were tentatively annotated based on accurate mass measurements, isotopic distribution patterns, retention behavior, and comparison of MS/MS fragmentation spectra with entries in the ReSpect database (negative ion mode). Compounds selected for docking were prioritized based on peak intensity and reported bioactivity.

### 3.4. Molecular Docking Studies

The protein–ligand docking studies were conducted with Molecular Operating Environment 2014 (MOE 2014; Chemical Computing Group, Montreal, QC, Canada) to investigate the binding of the selected phytochemicals to the active sites of gastric H^+^/K^+^-ATPase and COX-2. The molecular docking procedure was validated using the COX-2 crystal structure (PDB ID: 1CX2), yielding an RMSD value of 1.1023 Å, which is below the generally accepted threshold of 2.0 Å and confirms the reliability of the docking protocol. For H^+^/K^+^-ATPase (PDB ID: 5Y0B), classical redocking validation was not feasible because the selected crystal structure lacked a suitable co-crystallized ligand. Therefore, OMP was used as the reference ligand for comparative docking analysis. The selected phytochemicals were subsequently docked into the active sites of both COX-2 and H^+^/K^+^-ATPase, and their binding affinities and interactions were compared with those of the corresponding reference ligands.

The 2D structures of the investigated compounds were drawn using Chem Draw Ultra 16.0 software, then converted into 3D structures for geometry optimization. The compound structures’ energies were minimized using the MMF94FX force field with a gradient RMSD of 0.0001 kcal/mol. The structure of gastric PPi with the PDB code 5Y0B [69] and a selective COX-2 blocker with the PDB code 1CX2 [70] were downloaded from the Protein Databank (PDB). The 3D structure receptor was prepared by discarding water molecules and cofactors using MOE software. Subsequently, the ligands were docked at the binding site using the alpha triangle matching placement method. Refinement was performed using forcefield and scored using the affinity dG scoring system. The docking poses were visually inspected, and the pose exhibiting the lowest binding free energy and an RMSD value below 2 Å was selected.

### 3.5. Experimental Animals, Study Design, and Sample Collection

**Figure 13:**
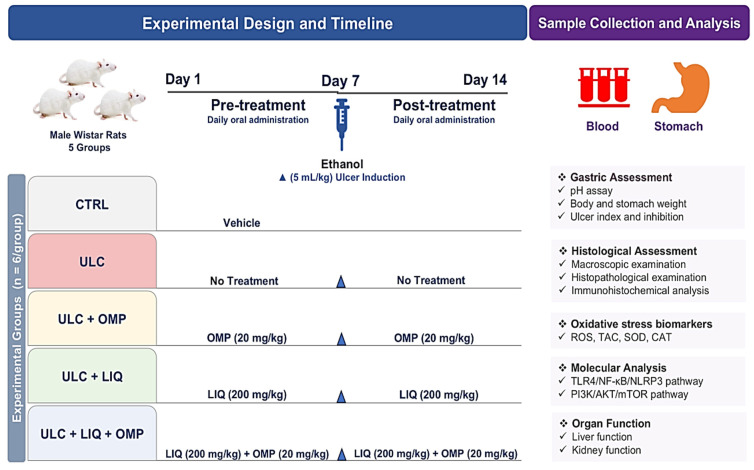
Schematic representation of the experimental design and study workflow used to evaluate the gastroprotective effects of LIQ extract and OMP against EtOH-induced GU in rats.

The study was performed on 30 healthy male Wistar rats, weighing 200–250 g. The rats were randomly allocated to five groups (*n* = 6 per group), as illustrated in Table 6 and Figure 13. All groups, excluding the CTRL group, were subjected to GU through oral gavage of absolute EtOH (5 mL/kg body weight) on day 7 [71]. The dosages of OMP and LIQ were determined based on previous studies [21,72,73]. Following the 14-day experimental period, rats were weighed and anesthetized with urethane (1.5 g/kg body weight, intraperitoneally). Blood samples were collected by cardiac puncture, after which the animals were humanely euthanized by exsanguination under deep anesthesia. Serum was separated by centrifuging at 3000 rpm for 5 min at 4 °C and stored at −20 °C until biochemical analysis. The stomachs were excised, rinsed with ice-cold physiological saline, and opened along the greater curvature, and the gastric juice was collected for gastric pH assessment. The gastric tissues were separated into two sections; one was fixed in neutral-buffered formalin for histological and IHC examinations, while the other was homogenized and stored at −80 °C for biochemical measurements [30].

### 3.6. Determination of Body Weight, Stomach Weight, Stomach Coefficient, and Gastric pH

The body weight of all experimental rats was recorded at the baseline and again at the end of the 14-day study period. The stomachs were excised, blotted dry carefully, and weighed using a calibrated analytical balance to determine absolute stomach weight. The stomach coefficient, an index of the relative stomach weight, was determined using the method outlined by El-Shinnawy et al. [74] as follows:

(2) $$\mathrm{Stomach}\;\mathrm{Coefficient}\;(\%)=\frac{\mathrm{Stomach}\;\mathrm{Weight}\;(g)}{\mathrm{Final}\;\mathrm{Body}\;\mathrm{Weight}\;(g)}\times 100$$

The gastric juice was collected, centrifuged at 3000 rpm for 5 min, and the pH of the resulting supernatant was measured using a calibrated digital pH meter (HI 110, Hanna Instruments, Smithfield, RI, USA).

### 3.7. Assessment of UI and Ulcer Inhibition

Gastric lesions were assessed and scored based on their severity, and the UI was calculated following the method outlined by Khan et al. [75]. Results were expressed as mean ± SD. The percentage of ulcer inhibition was calculated to evaluate the gastroprotective effect of therapies using the following formula:

(3) $$\mathrm{Ulcer}\;\mathrm{inhibition}\;(\%)=\frac{{\mathrm{UI}}_{\mathrm{ULC}}-{\mathrm{UI}}_{\mathrm{treated}}}{{\mathrm{UI}}_{\mathrm{ULC}}}\times 100$$

### 3.8. Macroscopic and Histopathological Examination of Gastric Tissue

The gastric tissues were subjected to gross (macroscopic) examination, and representative digital images were acquired to document the gross morphology of the gastric mucosa, including the presence, number, size, and distribution of GU [76,77]. The macroscopic images were subsequently used for qualitative comparison among the experimental groups. For histological evaluation, the gastric tissues preserved in 10% buffered formalin were dehydrated, embedded in paraffin, sectioned at 5 μm, and stained with hematoxylin and eosin (H&E). Histopathological alterations were assessed using a light microscope (Olympus XC30, Olympus Corporation, Tokyo, Japan) and semi-quantitatively scored according to a modified scoring system described by Al Asmari et al. [78]. Five randomly selected microscopic fields from each section were examined at ×100 and ×200 magnifications, and the mean histopathological score for each animal was calculated. Histopathological evaluation was performed by an investigator blinded to the experimental groups.

### 3.9. Biochemical Analysis

#### 3.9.1. Spectrophotometric Assay

Concentrations of urea (Cat. No. ab83362, UK) and uric acid (RayBiotech Inc., Peachtree Corners, GA 30092, USA) in serum were assessed. Serum creatinine levels and the activities of CAT and SOD in gastric tissue homogenates were assessed using Bio-Diagnostic kits (Bio-Diagnostic Co., 29 El-Tahrir St., Dokki, Giza, Egypt). Serum activities of ALT, ALP, and AST were measured using commercial test kits (Spectrum Diagnostics, Obour City, Cairo, Egypt) in accordance with the prescribed protocols.

#### 3.9.2. Enzyme-Linked Immunosorbent Assay (ELISA)

The concentrations of ROS (Cat. No. MBS039665), TAC (Cat. No. MBS1600693), NFkB (Cat. No: MBS453975), and NLRP3 (Cat. No. MBS7255410) in gastric tissue homogenates were determined by using rat-specific ELISA kits (MyBioSource Inc., San Diego, CA, USA) in accordance with the manufacturer’s guidelines. The levels of TLR4 in gastric tissue homogenates were measured utilizing (Cat. No. SEA753Ra; Cloud-Clone Corp., Wuhan, China) in accordance with the manufacturer’s protocol.

#### 3.9.3. RNA Extraction and Quantitative RT-PCR Analysis

Total RNA was extracted from gastric tissue using the Direct-zol™ RNA Miniprep Plus Kit (Cat. No. R2072; Zymo Research Corp., Irvine, CA, USA) following the manufacturer’s instructions. RNA concentration and purity were assessed using a Beckman spectrophotometer (Beckman Coulter, Brea, CA, USA). The mRNA expression levels of PI3K, AKT, and mTOR were determined by the SuperScript™ IV One-Step RT-PCR System (Cat. No. 12594100; Thermo Fisher Scientific, Waltham, MA, USA) as per the manufacturer’s instructions. Cycle threshold (Ct) values obtained were normalized using the housekeeping gene glyceraldehyde-3-phosphate dehydrogenase (GAPDH), and the relative gene expression was quantified using the 2^−ΔΔCt^ method. The primer sequences of the investigated genes are listed in Table 7.

### 3.10. IHC Examination

The IHC evaluation of stomach tissue was conducted to evaluate the expression of TNF-α, caspase-3, and PCNA using the methodology outlined by Saleh et al. [79]. The gastric sections were deparaffinized with xylene, rehydrated in progressively lower concentrations of alcohol, and incubated with 3% hydrogen peroxide. The sections were then treated with rabbit monoclonal antibodies targeting TNF-α (52B83, China), Caspase-3 (YPA 1086, China), and PCNA (BMA1034, China) as primary antibodies. Immunoreactivity was exhibited utilizing 3,3′-diaminobenzidine (DAB; Sigma-Aldrich, St. Louis, MO, USA). The IHC staining of TNF-α, Caspase-3, and PCNA was evaluated semi-quantitatively in ten randomly selected microscopic fields (×400 magnification) according to the protocol established by Hegazy et al. [80], utilizing color intensity and the proportion of positively stained cells to determine the immunoreactivity score (IRS).

### 3.11. Statistical Analysis

Statistical analyses were performed using GraphPad Prism (Version 10.5.0; GraphPad Software, San Diego, CA, USA). Normality and homogeneity of variances were evaluated using the Shapiro–Wilk and Levene’s tests, respectively. Data are presented as mean ± SD and analyzed using one-way ANOVA followed by Tukey’s post hoc test. Histopathological and IHC immunoreactivity scores, representing ordinal data, were analyzed using the Kruskal–Wallis test followed by Dunn’s post hoc test. Statistical significance was considered at *p* < 0.05. The sample size was determined a priori using G*Power software (Version 3.1.9.4; Heinrich Heine University Düsseldorf, Düsseldorf, Germany) according to the method described by Faul et al. [81]. The calculation was based on a one-way ANOVA design with an effect size (f) of 0.70, a significance level (α = 0.05), and a statistical power (1 − β = 0.80), indicating a minimum sample size of six rats per group (total *n* = 30). Correlation analyses were performed using IBM SPSS Statistics for Windows (Version 20.0; IBM Corp., Armonk, NY, USA). Spearman’s rank correlation analysis was performed to evaluate the associations among the investigated biomarkers. Correlation coefficients (ρ) and their corresponding significance levels were calculated using a two-tailed test.

## 4. Conclusions

The current findings revealed that LIQ extract considerably enhanced the therapeutic efficacy of OMP and provided greater gastroprotection against EtOH-induced GU. The combination treatment significantly decreased oxidative stress, inhibited TLR4/NF-κB/NLRP3-mediated inflammatory responses, and upregulated PI3K/AKT/mTOR gene expression, thereby supporting gastric mucosal integrity. LC-ESI-QTOF-MS/MS profiling further supported these findings by identifying several bioactive phytochemicals, and molecular docking studies showed favorable interactions with H^+^/K^+^-ATPase and COX-2, suggesting the potential role of LIQ in the observed gastroprotective effects. Collectively, these findings suggest that LIQ extract may serve as a promising adjunct to OMP for GU management. However, further studies are warranted to confirm the underlying molecular mechanisms.

## Figures and Tables

**Table 1:** Tentative identification of phytochemical constituents detected in LIQ extract by LC–ESI–QTOF–MS/MS.

Peak No.	Tentatively Annotated Compound	RT min	Observed m.z	Peak Area a.u.	Mass Error ppm	Adduct	Theoretical *m*/*z*	Formula
1	Glabridin	2.4	323.1002	5785.89	0.2	[M–H]^−^	323.02859	C_20_H_20_O_4_
2	Chlorogenic acid	2.188867	353.0085	8556.717773	1.4	[M–H]^−^	353.0878	C_16_H_18_O_9_
3	Quercitrin	4.391733	447.117	158,049.6563	−1	[M–H]^−^	447.09329	C_21_H_20_O_11_
4	Baicalein-O-glucuronide	6.3308	445.1373	494,240.4063	0.5	[M–H]^−^	445.07764	C_21_H_18_O_11_
5	Liquirtin	7.660217	417.1212	133,718	−0.8	[M−H]^−^	417.08273	C_20_H_18_O_10_
6	Kaempferol-O-pentoside (tentative)	9.14	417.122	86,788.58594	0.3	[M–H]^−^	417.08273	C_20_H_18_O_10_
7	Daidzein	9.4728	253.0522	256,153.375	−0.8	[M–H]^−^	253.05063	C_15_H_10_O_4_
8	liquiritigenin	10.0776	255.066	53,022.57422	1.71	[M–H]^−^	255.06556	C_15_H_12_O_4_
9	Daidzein-8-C-glucoside	10.50492	415.1053	5631.257324	0	[M–H]^−^	415.10345	C_21_H_20_O_9_
10	Luteolin	10.80073	285.0777	16,196.08105	0.1	[M–H]^−^	285.04047	C_15_H_10_O_6_
11	Formononetin	12.64397	267.0677	232,062.9531	−0.3	[M–H]^−^	267.06628	C_16_H_12_O_4_
12	Apigenin	12.72863	269.0823	9188.169922	0.6	[M–H]^−^	269.04553	C_15_H_10_O_5_
13	Naringenin	12.99295	271.0616	32,198.80273	1.3	[M–H]^−^	271.06119	C_15_H_12_O_5_
14	Quercetin-O-pentoside	13.10933	433.095	31,516.81641	−0.7	[M–H]^−^	433.07764	C_20_H_18_O_11_
15	Kaempferol-O-deoxyhexoside	14.14055	431.1017	33,592.4375	−0.6	[M–H]^−^	431.09836	C_21_H_20_O_10_
16	Glycyrrhizic acid	14.56187	821.4034	120,865.953125	0.1	[M–H]^−^	821.39648	C_42_H_62_O_16_
17	Glycyrrhetinic Acid/Enoxolone	19.3861	470.3405	420,641.844	1.91	[M–H]^−^	470.3396	C_30_H_46_O_4_
18	Licochalcone A	22.45	337.207	79,070.03	0	[M–H]^−^	337.05548	C_21_H_22_O_4_

**Table 2:** Molecular Docking Affinities of LIQ Extract Constituents Toward H^+^/K^+^-ATPase.

Compounds	Binding Affinity (kcal/mol)	Number of H-Bonds
OMP	−5.31	2
Quercetin-O-pentoside	−7.33	5
Glycyrrhizic acid	−8.04	5

**Table 3:** Molecular Docking Affinities of LIQ Extract Constituents toward COX-2.

Compounds	Binding Affinity (kcal/mol)	Number of H-Bonds
Bromo celecoxib	−6.21	2
Kaempferol-O-pentoside	−7.01	4
Apigenin	−7.14	5
Baicalein-O-glucuronide	−7.89	6
Glycyrrhizic acid	−8.20	6

**Table 4:** Effect of Treatments on UI and Ulcer Inhibition (%) in Experimental Groups.

	CTRL	ULC	OMP + ULC	LIQ + ULC	LIQ + OMP + ULC
Ulcer index	0.00 ± 0.00	19.65 ± 0.09 ^★^	16.64 ± 0.137 ^★●^	10.53 ± 0.033 ^★●^	1.65 ± 0.190 ^★●◆^
Ulcer inhibition (%)	0.00 ± 0.00	0.00 ± 0.00	16.46 ± 0.042 ^★●^	46.69 ± 0.171 ^★●^	90.61 ± 0.045 ^★●◆^

Data are presented as mean ± SD. Statistical analysis was performed using One-way ANOVA followed by Tukey’s post hoc test. Statistical significance was defined as *p* < 0.05. Symbols indicate comparisons as follows: (★) versus CTRL, (●) versus ULC, and (◆) versus OMP + ULC.

**Table 5:** Effect of Treatments on Liver and Renal Function Biomarkers in Different Experimental Groups.

	CTRL	ULC	OMP + ULC	LIQ + ULC	LIQ +OMP + ULC
ALT (U/mL)	32.93 ± 2.68	159.3 ± 3.38 ^★^	103.1 ± 2.70 ^★●^	126.4 ± 0.61 ^★●^	76.21 ± 1.33 ^★●◆^
AST (U/mL)	42.89 ± 1.47	181.7 ± 3.68 ^★^	126.8 ± 2.89 ^★●^	140.2 ± 0.28 ^★●^	84.32 ± 2.44 ^★●◆^
ALP (U/mL)	2.49 ± 0.44	8.08 ± 0.57 ^★^	4.71 ± 0.16 ^★●^	5.56 ± 0.12 ^★●^	3.80 ± 0.11 ^★●◆^
Creatinine (mg/dL)	1.74 ± 0.23	7.43 ± 0.21 ^★^	4.71 ± 0.025 ^★●^	5.72 ± 0.16 ^★●^	3.70 ± 0.27 ^★●◆^
Urea (nmol/mL)	2.71 ± 0.16	8.64 ± 0.32 ^★^	5.98 ± 0.11 ^★●^	7.00 ± 0.03 ^★●^	4.43 ± 0.32 ^★●◆^
Uric acid (mg/dL)	0.57 ± 0.02	3.71 ± 0.26 ^★^	1.99 ± 0.05 ^★●^	2.63 ± 0.12 ^★●^	1.07 ± 0.04 ^★●◆^

ALT, alanine aminotransferase; AST, aspartate aminotransferase; ALP, alkaline phosphatase. Data are presented as mean ± SD. Statistical analysis was performed using One-way ANOVA followed by Tukey’s post hoc test. Statistical significance was defined as *p* < 0.05. Symbols indicate comparisons as follows: (★) versus CTRL, (●) versus ULC, and (◆) versus OMP + ULC.

**Table 6:** Experimental design for EtOH-induced gastric ulceration in rats.

Group	Treatment Protocol
CTRL	Rats remained untreated throughout the experimental period.
ULC	Rats received a single oral dose of absolute EtOH on day 7 without further treatment.
ULC + OMP	Rats were orally pretreated with OMP (20 mg/kg/day) for 7 days before EtOH induction. EtOH was given 1 h after the last dosage of OMP on day 7 while OMP treatment continued to day 14.
ULC + LIQ	Rats were pretreated with LIQ extract (200 mg/kg/day, oral) for 7 days before EtOH induction. EtOH was delivered 1 h after the final LIQ dosage, and LIQ treatment was continued until day 14.
ULC + LIQ + OMP	Rats were pretreated with combined LIQ extract (200 mg/kg/day) and OMP (20 mg/kg/day) for 7 days before induction of EtOH. EtOH was provided 1 h after the last combination dose on day 7 and co-treatment continued until day 14.

**Table 7:** Forward and reverse primer sequences of the studied genes.

	Forward Primer	Reverse Primer
PI3K	ACACCACGGTTTGGACTATGG	GGCTACAGTAGTGGGCTTGG
mTOR	GACAACAGCCAGGGCGGCAT	ACGCTGCCTTTCTCGACGGC
AKT	AATGACCGGGGAGTCCGAAT	ATGTGCTTCATCCTGCCCAC
GAPDH	TGGATTTGGACGCATTGGTC	TTTGCACTGGTACGTGTTGAT

## Data Availability

The original contributions presented in this study are included in the article. Further inquiries can be directed to the corresponding author.
